# Network pharmacology-based mechanism prediction and pharmacological validation of Bushenhuoxue formula attenuating postmenopausal osteoporosis in ovariectomized mice

**DOI:** 10.1186/s13018-023-03696-7

**Published:** 2023-03-14

**Authors:** Chenjie Xia, Haowei Zhu, Jin Li, Hongting Jin, Danqing Fu

**Affiliations:** 1 Department of Orthopedic Surgery, Li Huili Hospital Affiliated to Ningbo University, Ningbo, 315048 People’s Republic of China; 2 Department of Orthopedic Surgery, Ningbo Yinzhou No. 2 Hospital, Ningbo, 315199 People’s Republic of China; 3 Institute of Orthopaedics and Traumatology, The First Affiliated Hospital of Zhejiang Chinese Medical University, Hangzhou, 310053 People’s Republic of China; 4 School of Basic Medical Sciences, Zhejiang Chinese Medical University, No. 548, Binwen Road, Hangzhou, 310053 Zhejiang Province People’s Republic of China

**Keywords:** Bushenhuoxue (BSHX) formula, VEGF, Angiogenesis, Postmenopausal osteoporosis, Network pharmacology

## Abstract

**Background:**

Bushenhuoxue (BSHX) formula, a ten-compound herbal decoction, is widely used to treat postmenopausal osteoporosis (PMOP) in China. However, the mechanism is not clear yet.

**Methods:**

The underlying biological processes and signaling pathways were predicted by network pharmacology. In vivo experimental study, 24 female C57BL/6 J mice were randomly divided into sham, ovariectomized (OVX) and BSHX formula groups. Mice in the latter two groups were subjected to bilateral ovariectomy, and mice in the BSHX formula group were extra treated by BSHX formula at an oral dosage of 0.2 mL/10 g for 8 weeks. The femur samples were harvested for tissue analyses including μCT assay, histology and immunohistochemical (IHC) staining of VEGF signaling.

**Results:**

A total of 218 active ingredients and 274 related targets were identified in BSHX formula. After matching with 292 targets of PMOP, 64 overlapping genes were obtained. GO and KEGG enrichment analyses on these 64 genes revealed that angiogenesis and VEGF signaling were considered as the potential therapeutic mechanism of BSHX formula against PMOP. Animal experiments showed that mice in the BSHX formula-treated group presented increased bone mass, microstructural parameters, blood vessel numbers and an activation of VEGF signaling (VEGF, COX2, eNOS and CD31) compared to the OVX mice.

**Conclusion:**

This study revealed that BSHX formula exerts anti-PMOP effects possibly through activating VEGF signaling-mediated angiogenesis.

## Introduction

Postmenopausal osteoporosis (PMOP) is a common bone disorder characterized by low bone mineral density and microstructure deterioration [1]. It is estimated that more than 15% of postmenopausal women over 50 years old are suffering in PMOP worldwide [2]. Most of them have low back pain, hunchback and fragility fractures in different degrees [3]. Although there exist a large amount of anti-osteoporosis drugs such as active vitamin D, estrogen receptor modulators, bisphosphonates and parathyroid hormone [4], various undesirable effects limit their application and efficacy [5]. Natural products and herbs attract increasing attention for their potential anti-osteoporosis effects and relative safety [6, 7].

Bushenhuoxue (BSHX) formula is a traditional herbal decoction composed of ten herbs (listed in Table 1). It has been widely used to treat various bone diseases in China for several decades, such as bone fracture [8], osteoarthritis [9, 10] and osteoporosis [6, 11]. The theory of “kidney governing bones” has well clarified that BSHX formula strengthens bone through tonifying kidney-qi [12]. In a recent study, we also found that BSHX formula can attenuate bone loss and bone structure destruction in ovariectomized (OVX) mice, confirming the anti-PMOP effects of BSHX formula [13]. However, its pharmacological mechanism remains unclear.

**Table 1:** The compositions of BSHX formula

Chinese name (abbreviation)	Botanical name	Family	Parts used	Proportion (%) (dosage, g)	Voucher specimen no.
Shu Di Huang (SDH)	*Rehmannia glutinosa* Liboscb.	*Scrophulariaceae*	Root	17.40 (9 g)	2001
Du Zhong (DZ)	*Eucommia ulmoides* Oliv.	*Eucommiaceae*	Bark	11.80 (6 g)	2002
Fu Zi (FZ)	*Aconitum carmichaeli* Debx.	*Ranunculaceae*	Root	11.80 (6 g)	2003
Gou Qi Zi (GQZ)	*Lycium barbarum* L.	*Solanaceae*	Fruit	11.80 (6 g)	2004
Rou Gui (RG)	*Cinnamomum cassia* Presl.	*Lauraceae*	Bark	5.90 (3 g)	2005
Shan Zhu Yu (SZY)	*Cornus officinalis* Sieb.	*Cornaceae*	Fruit	5.90 (3 g)	2006
Tao Ren (TR)	*Prunus persica* Batsch.	*Rosaceae*	Fruit	11.80 (6 g)	2007
Hong Hua (HH)	*Carthamus tinctorius* L.	*Asteraceae*	Corolla	5.90 (3 g)	2008
Shan Yao (SY)	*Dioscoreae opposite* Thunb.	*Dioscoreaceae*	Root	11.80 (6 g)	2009
Gan Cao (GC)	*Glycyrrhiza uralensis* Fisch.	*Leguminosae*	Root	5.90 (3 g)	2010

For the multi-component properties of TCM formula, conventional animal or cellular research strategies cannot meet the requirements to study massive molecular targets simultaneously. Network pharmacology is an emerging discipline that integrates pharmacology, bioinformatics, system biology and computer science [14, 15], which provides a systematic and integrative viewpoint to explore the relationships between TCM formula and disease [16–18]. Based on the research method of “Herb-Target-Gene-Disease,” network pharmacology can comprehensively predict the underlying molecular targets of BSHX formula in treatment of PMOP.

In the network pharmacology study, 64 overlapping genes between BSHX formula and PMOP were identified and their internal interactions were analyzed via a protein–protein (PPI) network. Furthermore, Gene ontology (GO) and Kyoto Encyclopedia of Genes and Genomes (KEGG) pathway enrichment analyses were performed on these overlapping genes to analyze the potential biological processes and singling pathways. According to the results of network pharmacology, an OVX mouse model was built to validate the above pharmacological mechanism.

## Materials and methods

### Preparation of BSHX formula and HPLC analysis

According to TCM theories, BSHX formula has functions of replenishing kidney, enriching essence and invigorating the circulation of blood. In this prescription, Shu Di Huang (SDH), Gou Qi Zi (GQZ), Shan Zhu Yu (SZY), Du Zhong (DZ), Fu Zi (FZ) and Rou Gui (RG) replenish kidney and enrich essence. Tao Ren (TR) and Hong Hua (HH) play the role in promoting blood circulation and removing blood stasis. All raw herbs in BSHX formula were provided by the First Affiliate Hospital of Zhejiang Chinese Medical University (Hangzhou, China). The plant materials were authenticated by Professor Ge, and the voucher specimens were preserved in Zhejiang Chinese Medical University. The extraction process of these herbs was performed as previously described [9]. *Eucommia ulmoides* Oliv Eucommiaceae*., Cornus officinalis* Sieb Cornaceae*., Glycyrrhiza uralensis* Fisch Leguminosae*., Aconitum carmichaeli* Debx Ranunculaceae*.*, *Lycium barbarum* L Solanaceae*.*, *Carthamus tinctorius* L Asteraceae*.*, *Rehmannia glutinous* Liboscb Scrophulariaceae*. and Dioscoreae opposite* Thunb Dioscoreaceae*.* were mixed and extracted with water at a ratio of 2:1:1:2:1:1:3:2. Other two herbs, *Cinnamomum cassia* Presl Lauraceae*.* and *Prunus persica* Batsch Rosaceae*.* were mixed at a ratio of 2:1 for ethanol extraction. These two parts were mixed and concentrated to 2 g crude drug/mL for intragastric administration.

In our previous study, high-performance liquid chromatography (HPLC) analysis was performed to preliminarily detect chemical ingredients of BSHX formula, and six major ingredients were identified, including loganin, amygdalin, pinoresinol diglucoside, liquiritin, cinnamaldehyde and hydroxysafor yellow A [9].

### Identification and target prediction of active ingredients

Traditional Chinese Medicine System Pharmacology Database (TCMSP™, http://lsp.nwu.edu.cn/tcmsp.php) and Traditional Chinese Medicines Integrated Database (TCMID, http://119.3.41.228:8000/) were used to search the chemical ingredients of BSHX formula. Oral bioavailability (OB ≥ 30%) and drug-like (DL ≥ 0.18) were set as the screening threshold [19]. UniProt database (https://www.uniprot.org/) was used to predict the target genes for these ingredients.

### PMOP targets screening

GeneCards Database (https://www.genecards.org/) and DisGeNET Database (https://www.disgenet.org/) were used to collect PMOP-associated target genes, with the screening filter of score > 10 for GeneCards Database and score > 0.1 for DisGeNET Database. Then, a Venn diagram was constructed to determine the overlapping genes between PMOP and BSHX formula, which were termed as the potential therapeutic molecular targets.

### Gene ontology and pathway enrichment analyses

The gene ontology (GO) enrichment analysis including biological process (BP), cellular component (CC) and molecular function (MF) terms was performed on the overlapping genes using DAVID database (version 6.8, https://david.ncifcrf.gov/home.jsp). KEGG database (https://www.kegg.jp/) was used to identify the potential signaling pathways.

### Herb-ingredient, PPI and target-pathway network construction

The network was constructed as follows: (1) The herbs of BSHX formula and its active ingredients obtained from TCMSP database and TCMID database were imported into Cytoscape3.8.0 software (http://www.cytoscape.org/) to establish a Herb-Ingredient network; (2) the overlapping targets were analyzed using the String database (https://string-db.org/) to build a PPI network. The PPI network topological feature was evaluated by three topological characteristics: degree centrality (DC), betweenness centrality (BC) and closeness centrality (CC); and (3) the overlapping genes and signaling pathway obtained from KEGG database were imported into Cytoscape3.8.0 software to establish a Target-Pathway network.

### Experimental groups and OVX model

Ten-week-old female C57BL/6 J mice provided by Shanghai Laboratory Animal Center of Chinese Academy of Science (Shanghai, China) were randomly divided into three groups (*n* = 8 in each group): the sham group, the OVX group and the BSHX formula group. Mice in the latter two groups were subjected to bilateral ovariectomy, whereas a sham surgery only extracting the equal surrounding fatty tissues of ovaries were performed in the sham ones. At the next day post-surgery, BSHX formula was orally administered to the mice in BSHX formula group at a dosage of 0.2 mL/10 g body weight every two days [13]. The mice in the sham group and the OVX group were given same dosage of 0.9% normal saline. All mice were killed 8 weeks after oral intervention. All animal experiments were approved by the Animal Ethics Committee of Zhejiang Chinese Medical University (LZ12H27001).

### μCT analysis

The femur samples were obtained from the mice in each group for micro-computed tomography (μCT) analysis. Three-dimensional (3D) images of femoral metaphysis were reconstructed using NRecon software. The parameters of bone microstructure including bone volume fraction (BV/TV, %), average trabecular number (Tb. N, 1/mm), average trabecular thickness (Tb. Th, mm), average trabecular separation (Tb. Sp, mm) and bone mineral density (BMD) were calculated as previously described [20].

### Histology and immunohistochemistry (IHC)

After μCT scanning, the femur samples were processed for paraffin sections at the thick of 3 μm as previously described [21]. Alcian Blue Hematoxylin/Orange G (ABH) staining was performed on these sections for morphological analysis. The numbers of blood vessel and trabecular area (%) in the region of interest were measured using OsteoMetrics software (Decatur, GA, USA) by two researchers. The IHC assay was detected as follows: (1) Sections were treated with 0.01 M citrate buffer (Solarbio, Beijing, CN) at 60 °C for 4 h as antigen retrieval; (2) sections then were incubated in primary antibodies of CD31 (Diagbio, AGR52748, CN), cyclooxygenase-2, (COX2, Huanbio, RT1159, CN), endothelial nitric oxide synthase (eNOS, Huanbio, R1412-3, CN) and vascular endothelial growth factor (VEGF, ARIGO, ARG10513, CN) overnight at 4 °C; (3) sections were incubated in secondary antibodies for 20 min and diaminobenzidine (DAB) solution for 1 min to detect positive staining; and (4) sections were counterstained with hematoxylin. ImageJ software was used to analyze the quantification of positive staining.

### Statistical analysis

All data were presented as mean ± standard deviation. Statistical analysis of unpaired Student's *t*-test (two groups) was performed with the software of SPSS 22.0. P^*^ < 0.05 was considered as statistical significance, and P^**^ < 0.01 was considered as highly statistical significance.

## Results

### Active ingredients of BSHX formula

**Fig. 1:**
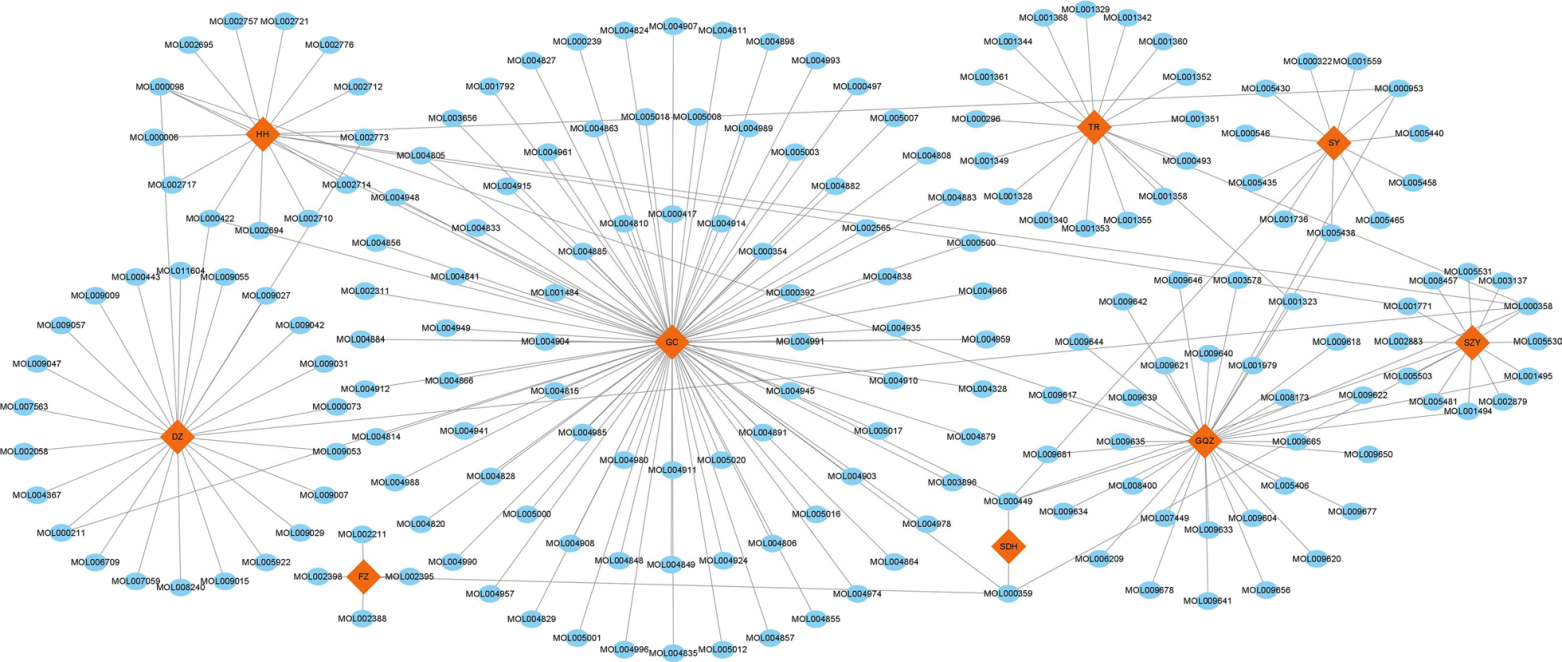
The network of Herb-Ingredient connection. The red square nodes represent herbs of BSHX formula including Shu Di Huang (SDH), Du Zhong (DZ), Fu Zi (FZ), Gou Qi Zi (GQZ), Rou Gui (RG), Shan Zhu Yu (SZY), Tao Ren (TR), Hong Hua (HH), Shan Yao (SY), Gan Cao (GC); the blue circles represent 218 active ingredients; the edges represent the direct relationship between herbs and active ingredients

After screening the TCMID database and TCMSP database with the ADME thresholds of OB ≥ 30% and DL ≤ 0.18, a total of 218 active ingredients were identified in BSHX formula, including 87 ingredients (40.0%) in GC, 36 (16.5%) in GQZ, 26 (11.9%) in DZ, 18 (8.2%) in TR, 17 (7.8%) in HH, 14 (6.4%) in SZY, 12 (5.5%) in SY, 5 (2.3%) in FZ, 2 (0.9%) in SDH and 0 (0%) in RG. As shown in Fig. 1, a Herb-Ingredient network was further constructed using Cytoscape software. According to the descending order of edge number in this Herb-Ingredient network, we listed and analyzed the top four ingredients that were beta-sitosterol (MOL000358, DL = 0.75, OB = 36.91, found in DZ, GQZ, HH, TR, SZY), stigmasterol (MOL000449, DL = 0.76, OB = 43.83, found in GQZ, SY, SDH, HH, SZY), quercetin (MOL000098, DL = 0.28, OB = 46.43, found in DZ, GC, GQZ, HH) and sitosterol (MOL000359, DL = 0.75, OB = 36.91, found in FZ, GC, SZY, SDH). Thus, these four ingredients are the potential material foundation of BSHX formula against PMOP.

### Target prediction and PPI network analysis

**Fig. 2:**
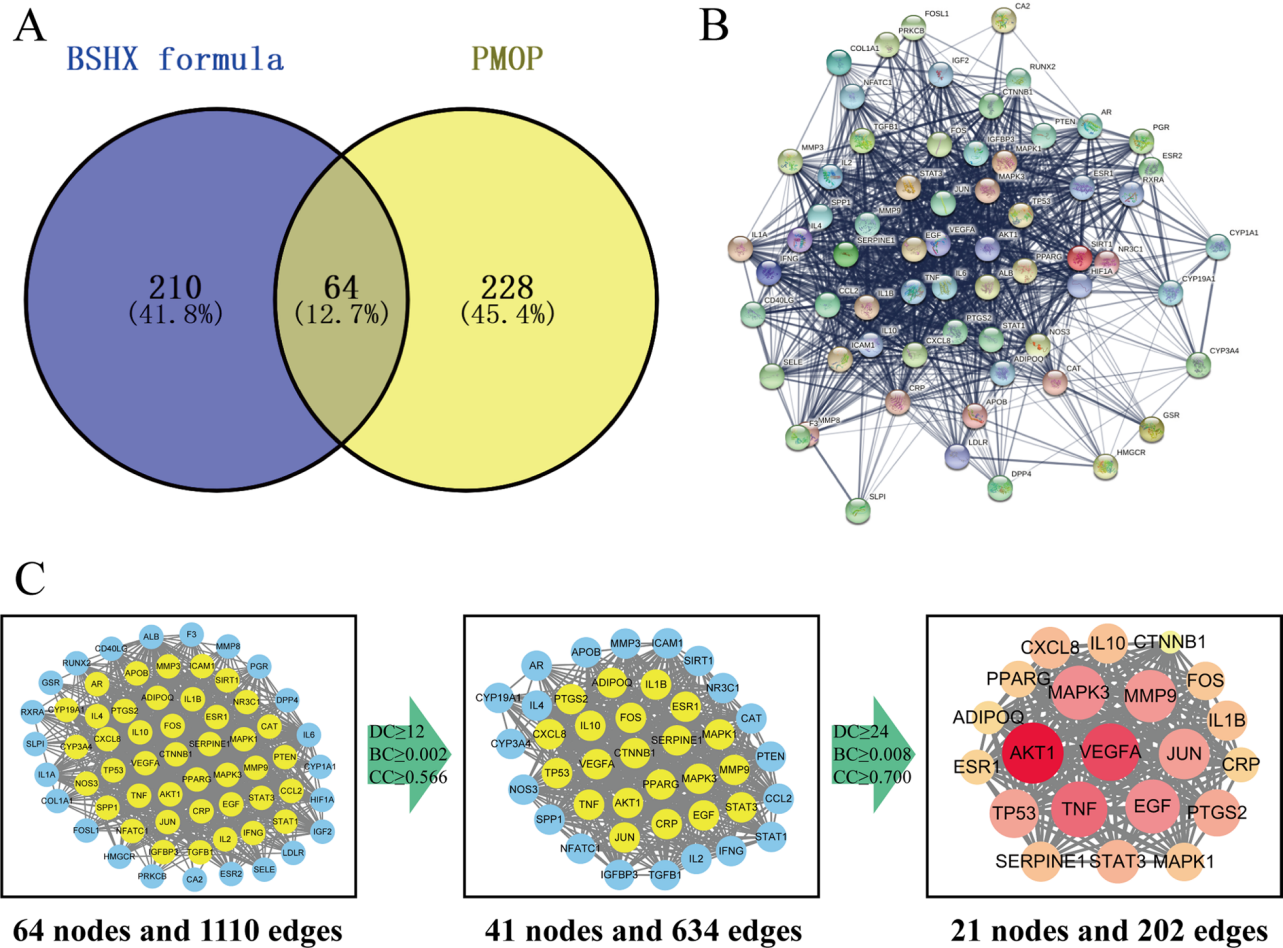
Venn diagram and PPI networks of 64 overlapping target genes between BSHX formula and PMOP. **A** Venn diagram identified 64 overlapping target genes between BSHX formula and PMOP. **B** a PPI network of 64 overlapping target genes was constructed from String database. Line thickness indicates the confidence level of the supporting data. **C** The topological screening process of PPI networks with the parameters of DC, BC and CC. In the third image, the bigger size and more brilliant color represent higher DC value. *DC* degree centrality, *BC* betweenness centrality, *CC* closeness centrality

Target fishing on the 218 active ingredients was conducted in Uniprot databases, and we obtained 274 related targets of BSHX formula among which there were 220 in GC, 206 in DZ, 201 in HH, 190 in GQZ, 67 in SY, 46 in TR, 51 in SZY, 10 in, FZ 29 in SDH. Then, 292 related targets of PMOP were obtained from GeneCards and DisGeNet databases. After establishment of Venn diagram, we identified 64 overlapping genes between BSHX formula and PMOP (Fig. 2A). These 64 overlapping genes were considered as the potential therapeutic targets.

A Protein–Protein Interaction (PPI) network was built on these 64 overlapping genes using the String database, which contained 64 nodes and 1110 edges (Fig. 2B). Then, we used three main parameters, “degree (DC),” “betweenness (BC)” and “closeness (CC),” as the screening thresholds to select the central target genes. After the first screening round of DC ≥ 12, BC ≥ 0.002 and CC ≥ 0.566, 41 nodes and 634 edges were obtained. Through the second screening round of DC ≥ 24, BC ≥ 0.008 and CC ≥ 0.700, only 21 nodes and 202 edges were identified (Fig. 2C). These hub targets played a more important role in the therapeutic effects of BSHX formula, and their information is listed in Table 2.

**Table 2:** Information of 21 hub targets

Uniprot ID	Gene symbol	Description
P31749	AKT1	RAC-alpha serine/threonine-protein kinase
P15692	VEGFA	Vascular endothelial growth factor A
P01375	TNF	Tumor necrosis factor
P27361	MAPK3	Mitogen-activated protein kinase 3
P14780	MMP9	Matrix metalloproteinase-9
P04637	TP53	Cellular tumor antigen p53
P05412	JUN	Transcription factor AP-1
P01133	EGF	Pro-epidermal growth factor
P35354	PTGS2	Prostaglandin G/H synthase 2
P40763	STAT3	Signal transducer and activator of transcription 3
P05121	SERPINE1	Plasminogen activator inhibitor 1
P22301	IL10	Interleukin-10
P01584	IL1B	Interleukin-1 beta
P10145	CXCL8	Interleukin-8
P35222	CTNNB1	Catenin beta-1
P37231	PPARG	Peroxisome proliferator-activated receptor gamma
P01100	FOS	Proto-oncogene c-Fos
Q15848	ADIPOQ	Adiponectin
P03372	ESR1	Estrogen receptor
P02741	CRP	C-reactive protein
P28482	MAPK1	Mitogen-activated protein kinase 1

### GO enrichment analysis and KEGG enrichment analysis

**Fig. 3:**
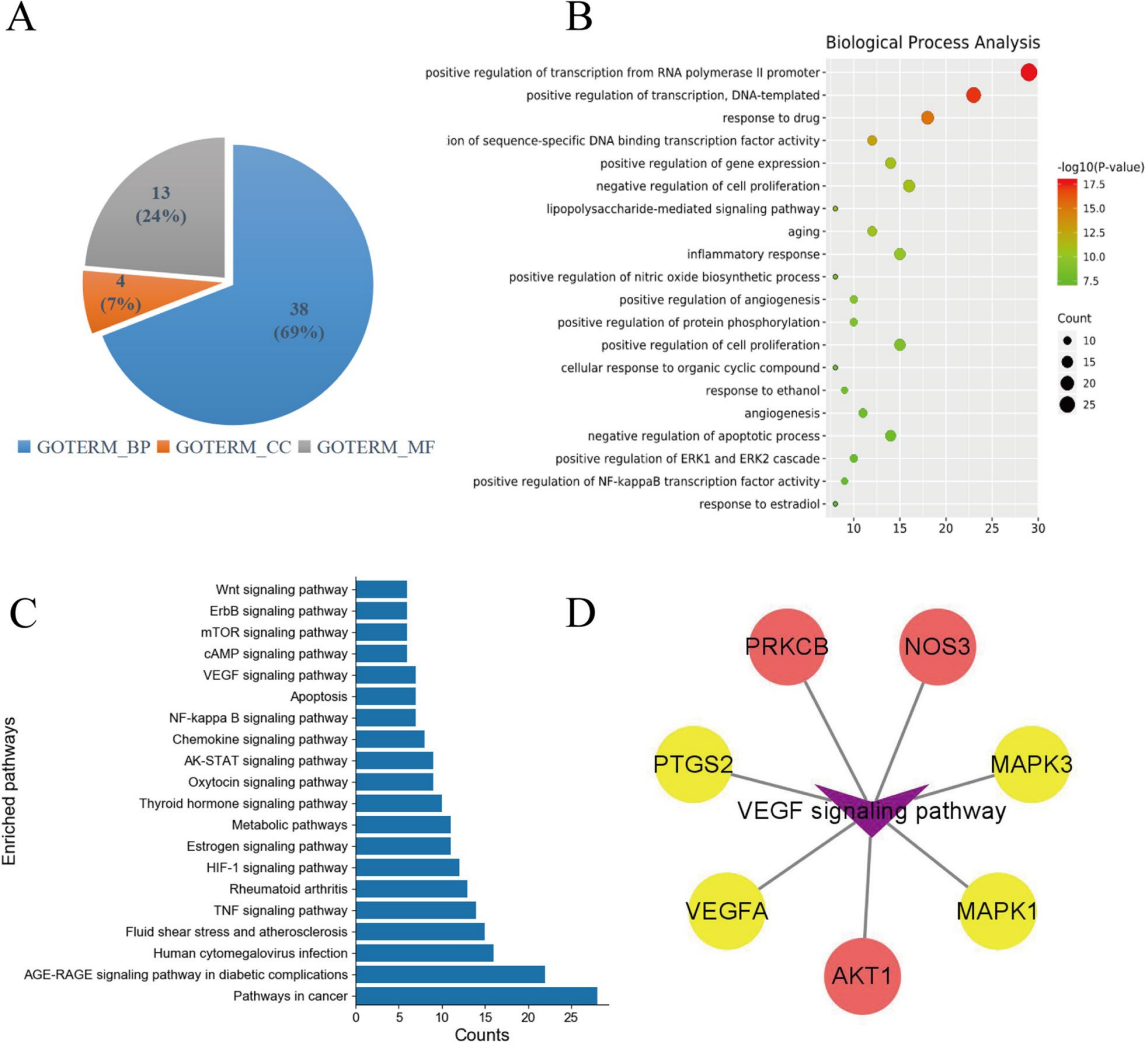
GO and KEGG enrichment analyses on 64 overlapping target genes. **A** The percentage of different GO items in DAVID database. Blue, GOTRIM_BP: biological process; yellow, GOTRIM_CC: cellular component; gray, GOTRIM_MF: molecular function. **B** The bubble diagram of BP items. Thirty-eight BP items were arranged in the descending order of *P*-value. **C** Details of the top 20 pathways obtained from KEGG database. **D** A total of 7 overlapping target genes including four hub target genes (VEGFA, PTGS2, MAPK1, MAPLK3) were enriched in VEGF signaling pathway. Yellow nodes: hub target genes; pink nodes: overlapping target genes

GO enrichment analysis was performed on these 64 overlapping genes using DAVID database. Based on the filter of FDR < 0.01, a total of 55 GO items were obtained, including 38 BP terms, 4 CC terms and 13 MF terms (Fig. 3A). As BP played a dominant role, we build a bubble diagram for the top 20 of them according to the descending order of log *P*-value (Fig. 3B). There were 6 BP terms concentrated into the category of angiogenesis, including positive regulation of angiogenesis (GO:0045766), angiogenesis (GO:0001525), cellular response to hypoxia (GO:0071456), positive regulation of endothelial cell proliferation (GO:0,001,938), response to hypoxia (GO:0001666) and positive regulation of vascular endothelial growth factor production (GO:0010575). Angiogenesis provides essential oxygen, nutrients as well as various bone cells for bone formation [22]. Thus, angiogenesis is a key biological process through which BSHX formula exacts anti-PMOP effects.

**Fig. 4:**
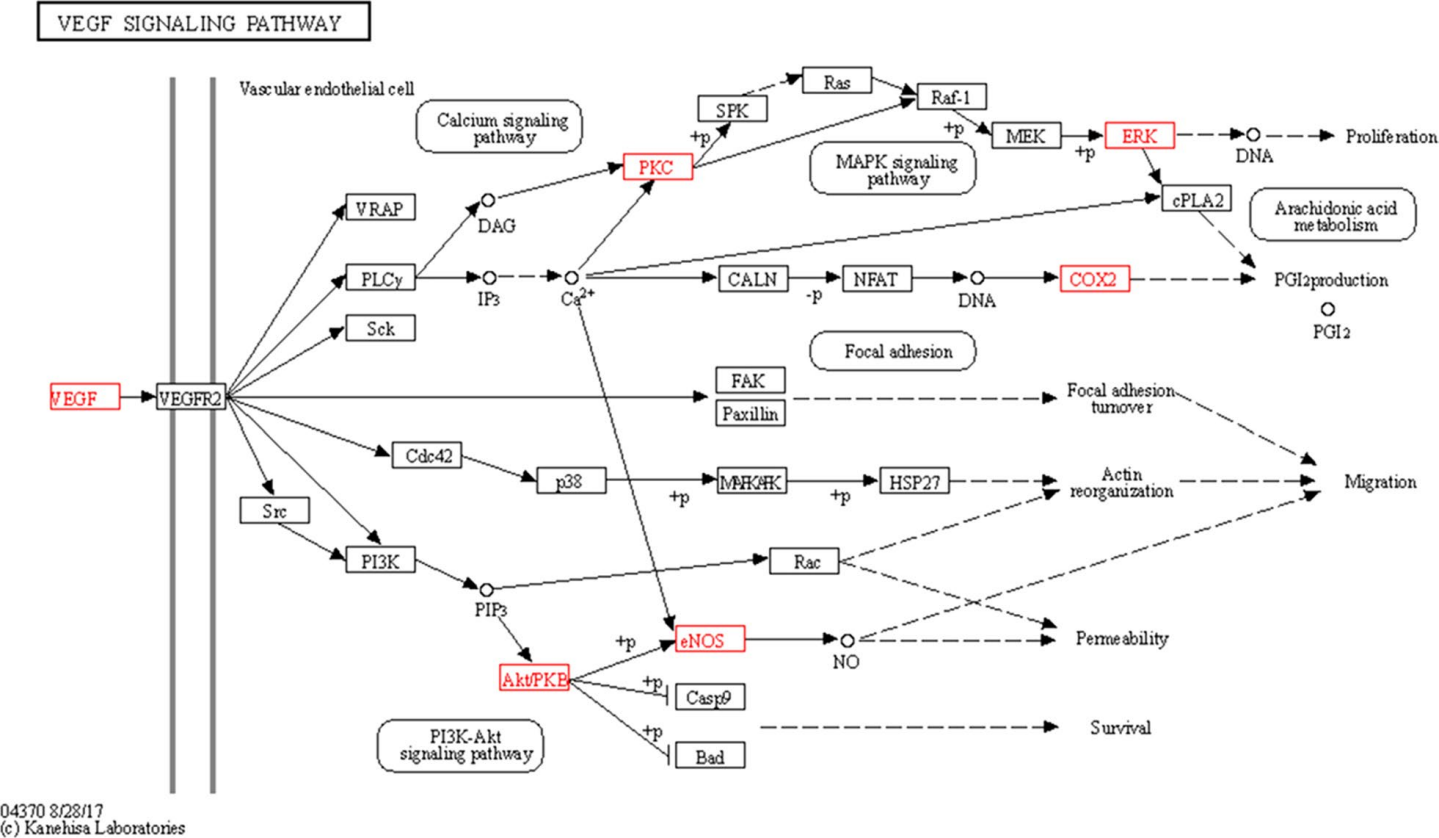
Seven overlapping target genes (red nodes) between BSHF formula and PMOP were enriched in VEGF signaling pathway. The signal transduction image from KEGG database showed that VEGF activates downstream targets including PCK, AKT, ERK, COX2, eNOS, etc., to promote vascular endothelial cell permeability, migration and proliferation for angiogenesis

To further determine the relevant pathways, KEGG enrichment analysis was conducted on the 64 overlapping genes. Based on the threshold of number ≥ 6, we screened a total of 99 pathways (20 of these listed in Fig. 3C), among which VEGF signaling pathway (hsa04370) directly regulates angiogenesis. A Target-Pathway network showed that seven overlapping genes, including four hub genes (VEGFA, MAPK1, MAPK3 and PTGS2), were enriched in VEGF signaling pathway (Fig. 3D). The signal transduction of VEGF signaling was obtained from KEGG database and is presented in Fig. 4.

### BSHX formula preserves bone mass in OVX mice

**Fig. 5:**
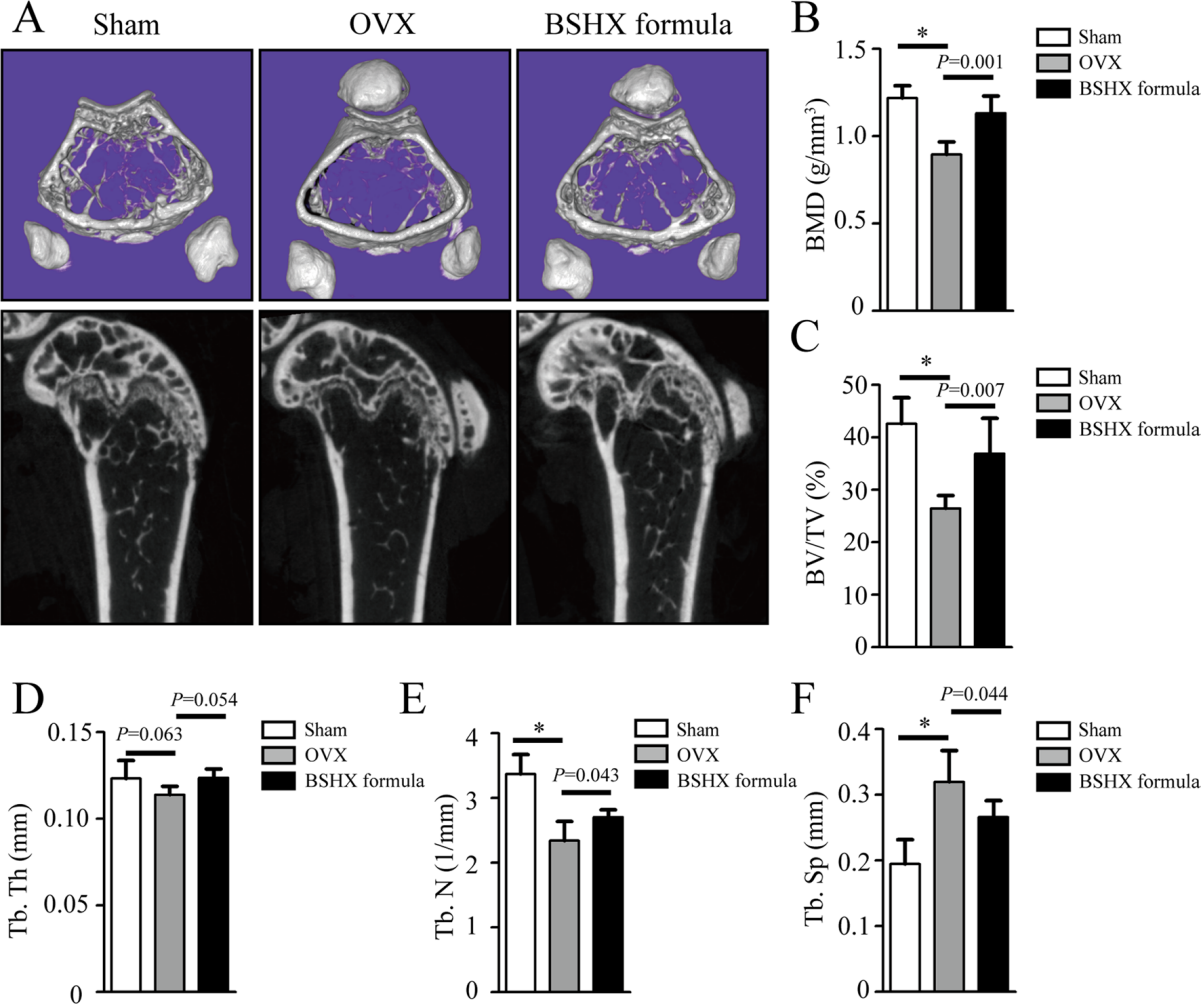
BSHX formula prevents bone loss in OVX mice. **A** Representative µCT images showed bone mass in the sham, OVX and BSHX formula-treated mice. The bone mass in BSHX formula-treated mice were significantly increased compared to the OVX mice. Quantification of microstructural parameters including BMD (**B**), BV/TV (**C**), Tb. Th (**D**), Tb. N (**E**) and Tb. Sp (**F**) in each group. The BMD, BV/TV and Tb. N were significantly increased, and Tb. Sp was significantly decreased in BSHX formula-treated mice compared to the OVX mice. *BMD* bone mineral density, *BV/TV* bone volume fraction, *Tb. N* average trabecular number, *Tb. Th* average trabecular thickness, *Tb. Sp* average trabecular separation. *P < 0.05, **P < 0.01

To carry out an animal experimental validation, C57BL/6 J mice were subjected to an OVX surgery and continuously treated with BSHX formula for 8 weeks. The 3D images of μCT showed severe bone loss in the OVX mice compared to the sham ones, and BSHX formula effectively alleviated bone loss (Fig. 5A). We also found that bone microstructure parameters were significantly improved after treatment of BSHX formula, including the increase of BMD, BV/TV, Tb.Th and Tb.N and the decrease of Tb.Sp (Fig. 5B–F). These results indicated that BSHX formula can preserve bone mass in the OVX mice.

### BSHX formula promotes angiogenesis in OVX mice

**Fig. 6:**
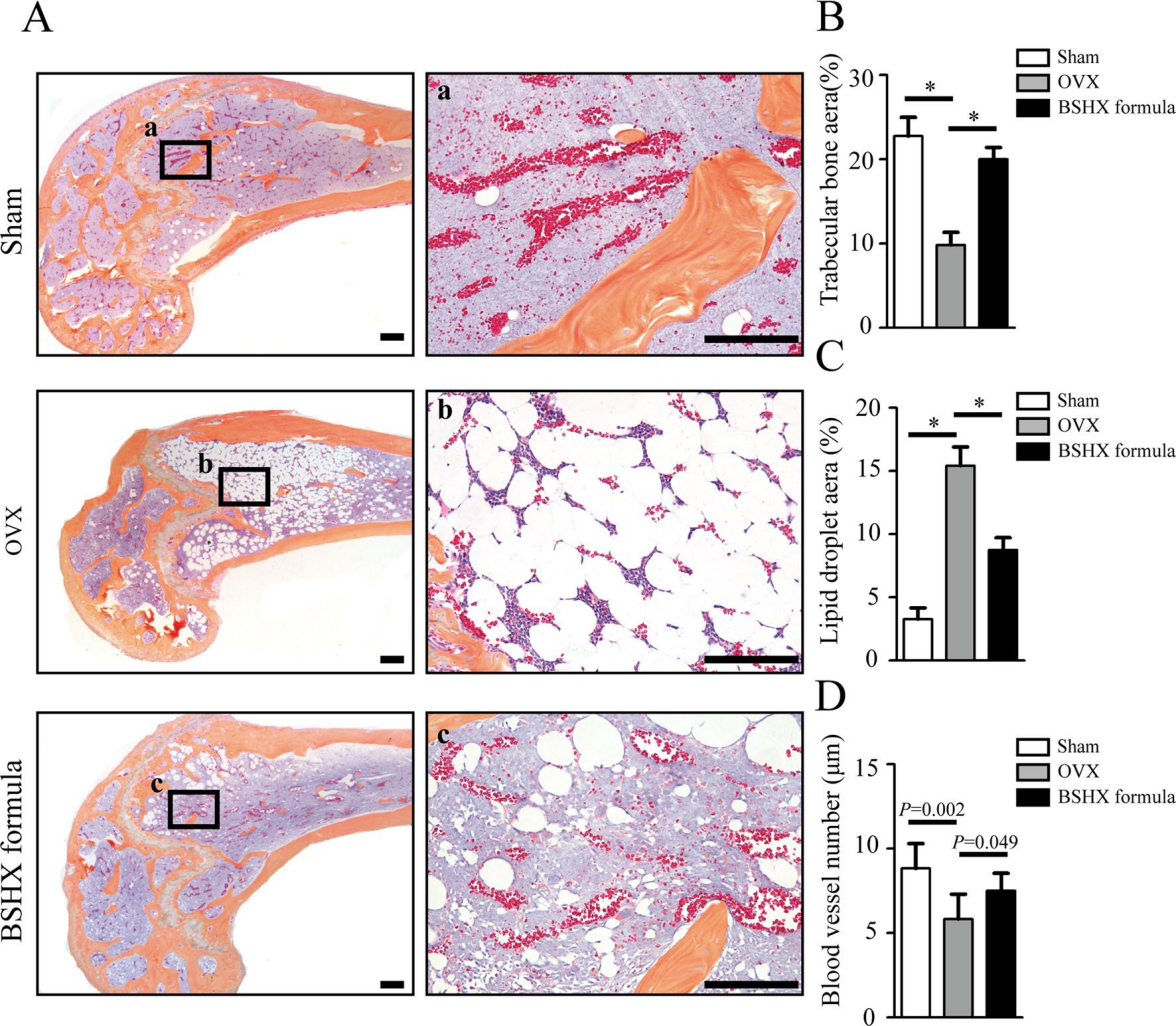
Changes of trabecular bone, lipid droplet and blood vessel in the OVX mice. **A** Alcian Blue Hematoxylin/Orange G (ABH) staining of distal femur. a–c: Boxed areas at a high magnification. Orange: trabecular bone; white: lipid droplet; red: blood vessel. **B** The area of trabecular bone (%). **C** The area of lipid droplet (%). **D** The number of blood vessel. Scale bars: 1000 µm. *P < 0.05, **P < 0.01

To verify the changes of angiogenesis, ABH staining was performed on the paraffin sections of each group, by which blood vessel can be dyed with red color. The representative images and quantitative analysis showed that thin trabeculae and massive lipid droplets in the OVX mice could be attenuated after treated with BSHX formula for 8 weeks (Fig. 6A–C), confirming the anti-PMOP effects of BSHX formula. ABH staining also revealed a significant decline in numbers of blood vessel in OVX mice compared to the sham mice, and BSHX formula effectively alleviated decreased blood vessels in the OVX mice (Fig. 6A, D). These findings indicated that BSHX formula can promote angiogenesis in the OVX mice.

### OVX-induced down-regulation of VEGF signaling is improved by BSHX formula

**Fig. 7:**
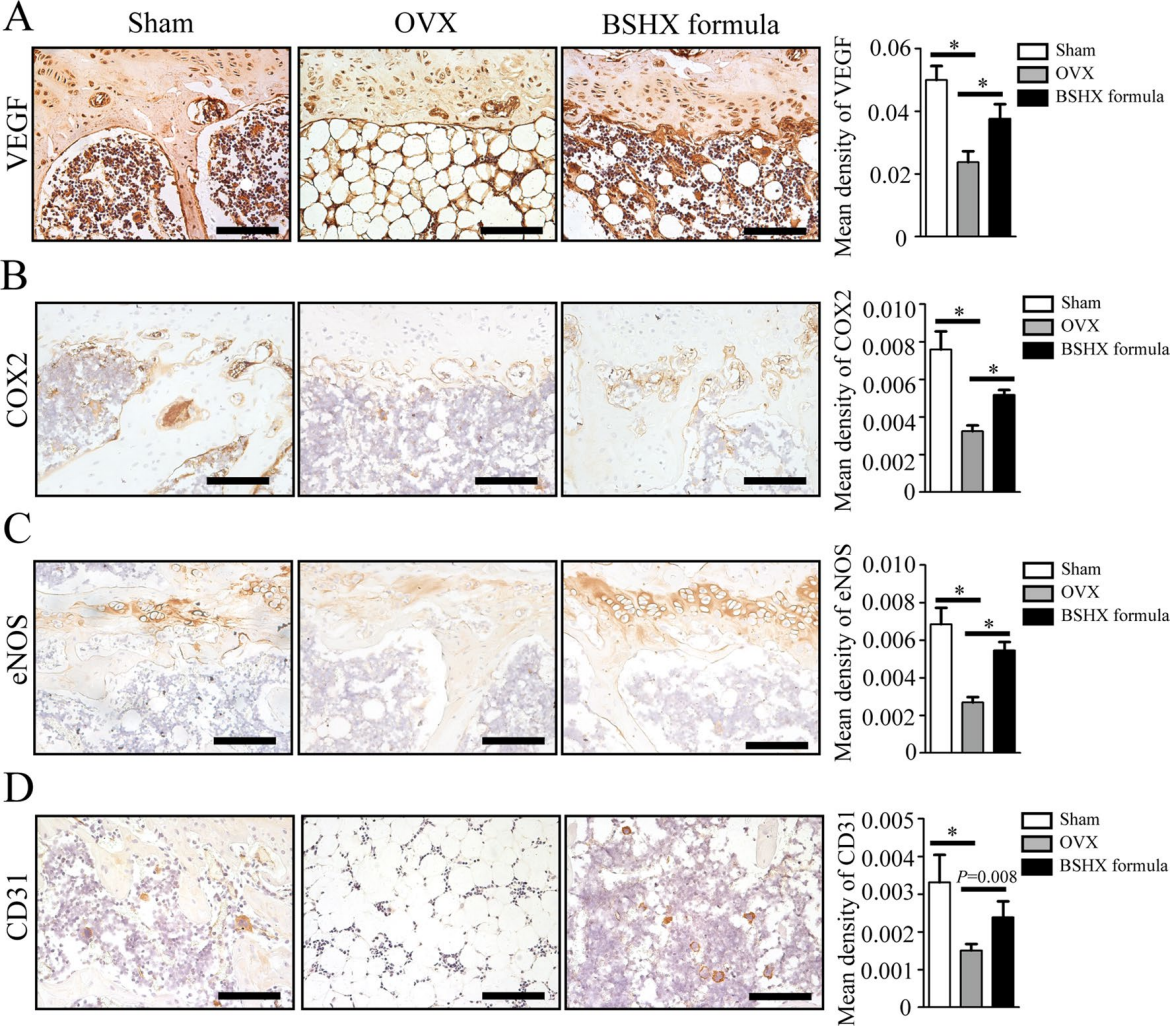
Down-regulation of VEGF signaling was restored by BSHX formula. Representative IHC images and qualification of VEGF (**A**), COX2 (**B**), eNOS (**C**) and CD31 (**D**). The expressions of VEFG, COX2, eNOS and CD31 were significantly decreased in the OVX mice compared to the sham mice. These changes of VEGF signaling were restored in OVX mice by BSHX formula treatment. Scale bars: 1000 µm. *P < 0.05, **P < 0.01

To determine the involvement of VEGF signaling pathway, we evaluated the expressions VEGF, COX2 and eNOS in each group by IHC staining. CD31, downstream target of VEGF signaling, is specifically expressed in vascular endothelial cells. The representative images and quantitative analysis showed that the levels of VEGF, COX2, eNOS and CD31 were significantly decreased in the OVX mice compared to the sham ones (Fig. 7A–D). But the OVX-induced down-regulations of VEGF, COX2, eNOS and CD31 were all restored after treatment of BSHX formula for 8 weeks (Fig. 7A–D), indicating that BSHX formula promotes angiogenesis possibly through activation of VEGF signaling.

## Discussion

With the progress of the aging population, PMOP has become a public health disease worldwide [23]. Currently, there is still lack of safety and effective anti-osteoporosis drugs. Natural products, especially Chinese medicine, have been largely studied to explore potential anti-osteoporosis drugs. A part of herbal compound (QingYan formula [24], Bu-Shen-Tong-Luo decoction [25], etc.) and single herb (eucommia ulmoides [26], herba epimedii [27], etc.) present anti-PMOP effects in OVX rats. In the present study, we aimed to explore pharmacological mechanism of BSHX formula through a network pharmacology-integrated animal experimental validation strategy.

According to the latest “network pharmacology evaluation method guidance” [16], 274 related targets of BSHX formula were obtained from TCMSP database and Uniprot database and 292 related targets of PMOP were obtained from GeneCards and DisGeNet databases. After screening the overlapping parts between BSHX formula and PMOP using a Venn diagram, we identified a total of 64 genes that were regarded as the potential therapeutic molecular targets. GO enrichment analyses based on these overlapping genes revealed a key biological process, angiogenesis. It is well known that angiogenesis is closely related to developmental and regenerative bone formation, named angiogenesis-osteogenesis coupling [28, 29]. Vascular invasion is a prerequisite for bone formation and mineralization [30]. Blood vessels provide essential oxygen, nutrients and endocrine hormones as well as the removal of waste products, acting as a bridge between bone and neighbor tissues [22, 31]. A specific subtype of blood vessel co-expressed with high CD31 and high endomucin help to generate an appropriate niche environment for osteoprogenitors [31]. In contrary, the reduction of blood vessels will contribute to the pathogenesis of various bone diseases, such as PMOP [32], femoral head necrosis [33] and fracture nonunion [34]. Thus, angiogenesis can serve as the therapeutic target for PMOP. Many medical and physical treatments have been report to alleviate osteoporosis in OVX animals through promoting angiogenesis [25, 35, 36]. In the subsequent experimental validation, we found that BSHX formula can effectively prevent bone loss caused by OVX. The results of histological staining showed a severe reduction of blood vessels in OVX mice, and the number of blood vessel were markedly increased after treatment of BSHX formula. All these data indicated that BSHX formula treats PMOP possibly through promoting angiogenesis.

KEGG analysis showed that VEGF, COX2 and eNOS are involved in VEGF signaling pathway. VEGF is one of the most important growth factors controlling angiogenesis [37]. It drives a series of well-orchestral angiopoietic events including proliferation and migration of endothelial cell, vessel sprouting, pruning and anastomosis by binding to VEGF receptors [38]. It has been reported that COX2 participates in VEGF-induced angiogenesis through activation of MAPK signaling [39]. Moreover, as the synthetic rate-limiting enzyme, COX2 can regulate bone formation by controlling synthesis of prostaglandin E2 [40]. Conditional knockout of COX2 gene causes severe osteoporosis in the mice [41]. eNOS has been found to promote migration and proliferation of endothelial progenitor cells for angiogenesis [42]. In addition, eNOS plays a key role in regulating osteoblast activity and inhibiting bone resorption [43]. CD31, a specific marker of vascular endothelial cell, can reflect the progress of angiogenesis [44]. In the animal experimental validation, we found that the expressions of VEGF, COX2, eNOS and CD31 were inhibited in the OVX mice, but their down-regulations caused by OVX were significantly improved by BSHX formula. Combined with the improvement in angiogenesis in BSHX formula-treated mice, it can be concluded that BSHX formula exerts anti-PMOP possibly through VEGF-mediated angiogenesis.

There are several limitations in the present study. First and foremost, network pharmacology is a discipline of calculation and prediction; it is bound to have some false positives in ingredients, biological processes and signaling pathways. Secondly, we only screened 218 active ingredients of BSHX formula in TCMSP database, which might leave out a part of active ingredients and targets of BSHX formula. In addition, pharmacokinetic analysis is still needed for determination of the exact ingredient(s). Thirdly, GO and KEGG enrichment analyses revealed numbers of underlying biological processes and signaling pathways when BSHX formula treats PMOP, while only angiogenesis and VEGF signaling were verified in animal experiments. Finally, cellular experiments or a rescue design in animal study with a signal inhibitor would further demonstrate the important role of VEGF-mediated angiogenesis for BSHX formula against PMOP.

Overall, network pharmacology comprehensively analyzed the potential therapeutic molecular targets, biological processes and signaling pathways of BSHX formula against PMOP. The subsequent animal validation experiments revealed that BSHX formula exerts the anti-PMOP effects mainly via VEGF-mediated angiogenesis. Network pharmacology followed by experimental validation is an effective and reliable research pattern for pharmacological mechanism of Chinese medicine.

## Data Availability

The data used to support the result of this study can be obtained from the corresponding author.
